# Efficacy of a volatile pyrethroid spatial emanator (SE) in reducing *Anopheles* host-seeking in outdoor kitchens in Southern Zambia

**DOI:** 10.1371/journal.pone.0335514

**Published:** 2025-11-06

**Authors:** Timothy A. Burton, Limonty Simubali, Lewis Hambayi Kabinga, Justin Moono, Pebble Moono, Jennifer C. Stevenson, Monicah Mirai Mburu, Edgar Simulundu, Neil F. Lobo

**Affiliations:** 1 Eck Institute for Global Health, University of Notre Dame, Notre Dame, Indiana, United States of America; 2 Macha Research Trust, Macha, Southern Province, Zambia; 3 Department of Molecular Microbiology and Immunology, Bloomberg School of Public Health, Johns Hopkins University, Baltimore, Maryland, United States of America; Al-Azhar University, EGYPT

## Abstract

**Introduction:**

In southern Zambia, malaria transmission is low, with outdoor biting *Anopheles* mosquitoes playing a significant role in malaria transmission. Locals cook in outdoor, open-walled kitchen shelters, exposing them to these outdoor biting vectors and malaria. Volatile pyrethroid spatial emanators (SE) operate through a mode of action which could provide local protection around these kitchens. In this study, SE devices containing the pyrethroid transfluthrin were deployed to local kitchens, where human landing collection (HLC) was utilized to determine differences in host-landing rates associated with protection.

**Materials and Methods:**

Forty-one households from two villages were enrolled in the study in clusters of five (or six) and randomly assigned a treatment by cluster. Local mosquito collectors were recruited and trained to conduct HLCs from 18:00–06:00 once per week for 15 weeks. SE and placebo devices were replaced monthly. Following collection, mosquitoes were returned to the lab for morphological identification. Results were analyzed in R with negative binomial generalized linear mixed models (GLMM) considering all-night and per-hour capture.

**Results:**

A total of 3021 mosquitoes were collected during the follow-up period, with *Anopheles* species composing roughly half of all specimens. *Anopheles* activity was lower in SE protected kitchens based on all night and hourly comparisons. Mosquito activity was highest in the middle of the night, and SE was not significantly associated with protection between 18:00–21:00. SE usage appeared to reduce mosquito host-seeking activity by approximately 65–70%, with this efficacy appearing to diminish gradually over time to approximately 20–25% four weeks after opening, at which point they were replaced. Culicine mosquito behavior was not significantly impacted by the SE.

**Discussion:**

The SE device provided protection to individuals within protected kitchen structures overnight and during most hours of the night. The number of mosquitoes was lowest during the early collection hours between 18:00–21:00, a period in which the SE devices did not significantly impact mosquito host-seeking behavior. This result has implications for this use-case and should be further explored.

## Introduction

Reductions in malaria transmission have been primarily attributed to vector control through the dissemination of interventions throughout the endemic world [1]. Long lasting insecticide treated nets (LLIN) and indoor residual spraying (IRS) are the only primary interventions currently approved by the WHO for controlling populations of *Anopheles* mosquitoes which transmit malaria, specifically targeting indoor feeding and resting behaviors [2]. As distribution and usage have become more widespread, local vector bionomics have frequently been observed to adapt in response, with rising rates of resistance towards the active insecticide and behavioral shifts towards biting times and locations wherein individuals are less protected [3–11]. These shifts have been observed within a species, with shifts in species compositions within a sympatric population towards species which display these behaviors [12,13]. This has contributed to the proportional rise in behaviors such as outdoor biting, or earlier or later biting when people are not under LLINs, and their contribution to transmission [8,9,14,15]. Behaviors and transmission in these spaces and times must be addressed to enable malaria control efforts to sustain progress and continue towards local elimination.

Outdoor and early evening biting have been observed to maintain low levels of transmission in areas where bed net usage is high [3,5,6,8]. In some cases, this has contributed to rebounding malaria as the original vector populations shift in response to intervention deployment and usage, sometimes exacerbated by a reduction in overall intervention pressure as incidence rates fall [16]. In Zambia’s Southern Province – the site of this entomological study – malaria reduction efforts have successfully focused on indoor biting and resting behaviors to reduce the burden of malaria to a level where it is now considered a near-elimination zone. LLIN coverage in the area is high although usage is thought to be low, with low or no IRS coverage in the area. Presently, malaria transmission is low and seasonal with local vector populations displaying a considerable amount of outdoor and early evening/late morning biting [17–19]. The predominant vectors in the area have generally been reported to be the outdoor-biting *An. arabiensis* and *An. squamosus* [17,19–21].

The overlap of local human and vector behaviors influences where and when disease transmission is likely to be taking place, and thus the deployment of interventions to spaces where these behaviors meet [22,23]. In many endemic areas, humans spend considerable amounts of time outdoors, and spaces where people congregate or spend a lot of time, such as the peri-domestic space, are usually unprotected by LLINs and IRS. This space can include outdoor kitchens, shelters, animal enclosures, clearings, etc., depending on local customs. Mosquito behavior can also be variable between different transmission settings or locations, and a large knowledge gap exists regarding vector behaviors in many areas [24]. Understanding local vector behaviors and crafting interventions which can target the overlap of these behaviors with the local human behaviors can help to close existing gaps in protection and more thoroughly interrupt the disease transmission cycle.

Spatial repellents/emanators are a promising tool for controlling mosquito vectors in a variety of locations, since they are often easily deployable, simple to use, and not obtrusive to daily life. Spatial emanators specifically rely on passive emission – without an active power source – of an insecticide active ingredients which disrupt the ability of mosquitoes to successfully host-seek and probe for blood, and were given a conditional recommendation in the August 2025 WHO guidelines for malaria [25,26]. Spatial repellents which utilize volatized compounds are theorized to create a zone of protection around the device, with the degree of protection varying by distance from the chemical source. Evidence is generally limited regarding the area of protection, which likely depends on the type of product and numerous local environmental factors. This study presents a field evaluation of a volatile pyrethroid spatial emanator (SE) product which was designed to prevent outdoor mosquito biting activity in the vicinity. It contains transfluthrin as an active ingredient, a pyrethroid which has reduced the biting activity of *Anopheles* and *Aedes* mosquitoes in laboratory and field trials [27–35]. This effect has consistently been observed in multiple different transfluthrin-based products against laboratory-raised and/or field-captured mosquitoes resistant to other pyrethroids such as permethrin or deltamethrin [32,36,37]. The efficacy of the SE product evaluated in this trial was previously observed for at least four weeks after opening in a semi-field environment at the same study site, later corroborated by evidence from multiple field trials in Indonesia [38–40]. This trial represents the first field trial of the device in the low transmission setting of southern Zambia and seeks to establish the efficacy of the device as an intervention against outdoor biting mosquito species in outdoor kitchen structures.

## Materials and methods

### SE description and deployment

**Fig 1:**
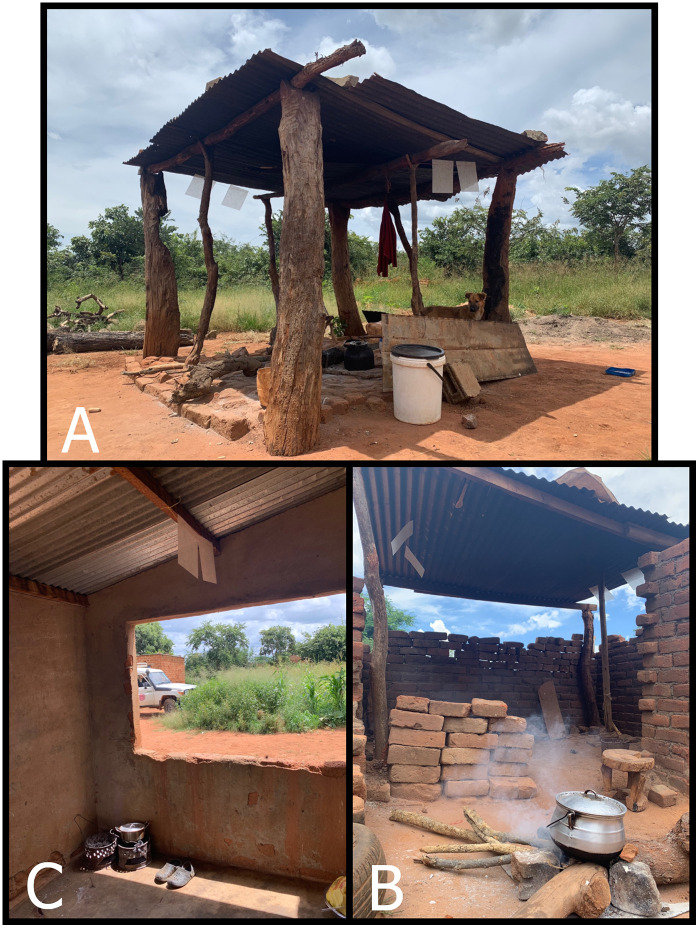
SE deployed in three treatment structures representative of kitchens in the area containing no walls (A) or open walls (B and C).

The SE (spatial emanator) is a passive, transfluthrin based spatial emanator. The tested devices are a prototype version of the PIC® BITEBARRIER®, designed to release transfluthrin slowly over the life of the product with a recommended lifetime of 21 days. Each device consisted of 1.5g of transfluthrin total in two thin 30x30cm sheets and can be easily hung from the eaves or rafters of local kitchen structures to afford protection from the elements while also being less disruptive to normal activities. The manufacturer recommends one device for closed areas of similar size to the outdoor study kitchens. During the study, two devices (four “sheets”) were deployed to the outdoor treatment kitchens, where they were installed on opposite sides (one device, or two sheets on each side) roughly 1.5m from the ground (roof height, varying slightly by structure) (Fig 1). Simple placebos were constructed from roughly equivalent sized sheets of plastic. Participants were not informed of enrollment into treatment or control arms, but the control “device” was not a true placebo (i.e., it was made from different material, rather than a no-chemical version of the SE). Thus, the study was not blinded. Treatment and placebo devices were assigned to study kitchens for the duration of the study period and were replaced every four weeks (roughly 28 days).

### Study site

**Fig 2:**
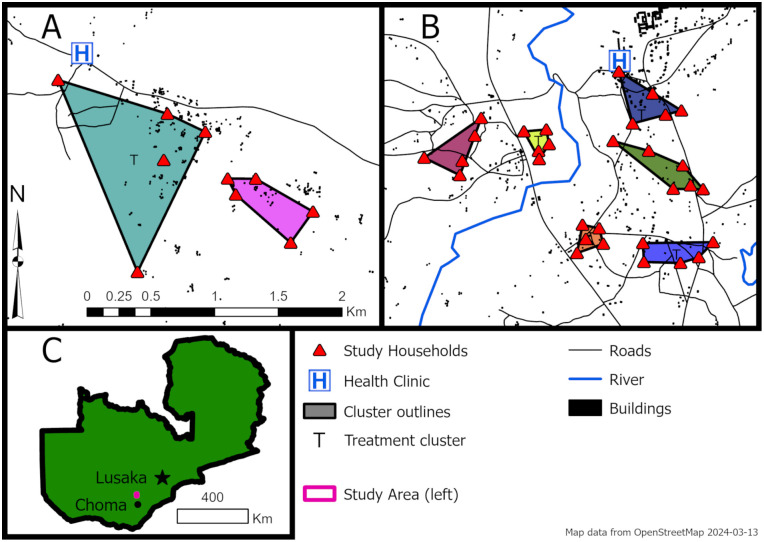
Location of study site clusters in Nalube (A) and Mapanza (B), and the general study site location in Southern Province, Zambia (C). Building locations, roads, and rivers were displayed using data from OpenStreetMap, available under the Open Database License.

The study was conducted in Southern Province, Zambia, in an area that is broadly characterized as a Miombo woodland. The region typically experiences three seasons, with the rainy season occurring between November and April followed by a cool dry season through July before a hot dry season through October. Two villages were selected for the study based on historical entomological and epidemiological data indicating that *Anopheles* mosquitoes were present and active. Enrolled households were selected based on previous collection data and a distance of 100m from other enrolled study households. Thirty-one households were enrolled from Mapanza village, situated at the intersection of a main road and moderately sized river. The remaining ten households were enrolled from the more remote and less densely populated village of Nalube. Households were enrolled in eight clusters of five (one cluster of six) which were generally located near each other (Fig 2). Clusters were assigned to the intervention or control arm via a series of coin flips.

### Study design

A total of 41 households across a total of eight clusters were enrolled in the study. Twenty were designated as treatment kitchens with the remaining twenty-one assigned as controls, and all households were followed for thirteen weeks after deployment of the SE devices. Collections occurred in each household kitchen once per week, on Monday through Thursday nights. Each of these nights, mosquito collections occurred in ten (on one night, eleven) kitchens from two adjacent clusters (one treatment and one control).

### Study participants

Households were contacted during the enrollment period in the early transmission season via a visit from study staff. Consent forms were translated into the local language and read to heads of households, with English consent forms also provided upon request. Consenting household heads were asked to identify two adult males who they would trust to spend nights in their kitchen collecting mosquitoes. These individuals (82 in total) provided informed consent and were trained to conduct HLC in study kitchens as pairs. Collectors stayed assigned to the same kitchens for the duration of the study without rotation. Five community health workers (two from Nalube and three from Mapanza) were enrolled as local supervisors to travel between households confirming collections were taking place and identifying equipment failures. Collectors and supervisors were provided compensation and given Deltaprim as malaria prophylaxis during the study period. Deltaprim was used because it is the required prophylaxis in Zambia, available locally and accepted by the community. Participants were advised on the risks and told to report any reactions to the drug, where their place in the study would be safe and they would be moved to a different prophylaxis. An additional small field team coordinated collections, dispensed and recovered collection materials, conducted spot checks during overnight collections, and secured data.

### Mosquito collections

Human landing collections (HLC) were employed for all mosquito collections during the study period [41]. Enrolled collectors were trained in group training sessions at the local health clinic, where they were instructed on proper aspirator technique and data recording. Trained entomologists joined the new collectors in the field on their first collection nights to ensure proper technique and adherence to the protocol. Collections occurred from 18:00–05:45 on given nights using mouth aspirators (Hock Company, USA). Collectors were provided with head torches, paper collection forms, and pencils. Collections occurred for the first 45 minutes of each hour prior to a 15-minute break, with mosquitoes placed into separate cups based on the hour of collection. Once filled, cups were kept on tables or elevated surfaces within the kitchens. Mosquitoes were brought immediately to the lab and frozen (killed) before counting and identification in the lab. Mosquitoes were counted by household, and collection forms included only household number and collection hour to minimize observation bias. Mosquitoes were identified morphologically to species using the standard key for the region developed by Maureen Coetzee and updated in 2020 [42]. Ten households (two clusters) were active on each night, with collections occurring on four nights per week and each household revisited weekly. To prevent the spread of COVID-19 and other possible infections, two complete sets of aspirators were used to allow for thorough cleaning and disinfecting between uses. Thus, each collector received a disinfected aspirator on each night.

### Study period

The enrollment period began on January 24, 2022, with training conducted in groups of twenty collectors over the course of four separate sessions. Enrollment was completed by February 18, 2022. Field training collections were conducted in each household kitchen after training, with each household undergoing field training once before devices were deployed to kitchen structures. SE and control devices were first distributed on February 21, with the final kitchen receiving them on March 9. The replacement period was determined from the date of the first deployment at each household. After the start, the normal schedule of weekly visits was adhered to with a few exceptions. Thirteen additional follow-up collections occurred in each household kitchen, weekly from Monday through Thursday night (two clusters each night) until early June.

### Additional data collection

A short survey of study kitchens was conducted to determine basic characteristics: the size of the kitchen, construction material, style, and the number of household occupants including the number of bednet users. During collection nights, collectors were asked to record additional information each hour. This included the number of other individuals and animals (noted separately) occupying the structure during the hour. This measurement was used to enhance secondary analysis which overlaps human and mosquito behaviors to evaluate “true” risk of exposure to *Anopheles* biting associated with using the outdoor kitchens. Additionally, the presence of a small cooking fire and rainfall was indicated for each hour since these factors may influence mosquito activity. Weather recordings from the Macha Research Trust campus were recorded every 15 minutes using an Onset HOBO USB Weather Station and calculated into hourly and nightly mean values. Satellite imagery generated from two single Planet Labs, Inc. captures on Feb 26, 2022 and May 05, 2022 by the PSB.SD (“PSBlue Super Dove”) instrument [43] were used to calculate normalized difference vegetation index (NDVI) for the study area; focal averages of NDVI across a range of distances were input into models.

### Data verification

Collection forms received from HLC collectors were input into digital forms and compared to the mosquito counts generated from laboratory processing of the mosquitoes using a simple R script. This acted as a quality control verification and the numbers were generally well aligned. On occasion there were small discrepancies, most likely due to mosquitoes escaping during transport from the field to the lab or collection of non-mosquito species. The number confirmed during lab processing was used for statistical analysis since these were identified as *Anopheles* by a trained professional entomologist.

### Data analysis

Following verification, data was cleaned and analyzed in R version 4.2.2. Data cleanup, summarizing, and plotting utilized the ‘tidyr’, ‘dplyr’, and ‘ggplot2’ packages, respectively. Generalized linear mixed models were used to describe the *Anopheles* and non-*Anopheles* count data, using a negative binomial underlying distribution and a logarithmic link function after observing overdispersion. Two primary models were generated, differing by their response variable of nightly or hourly *Anopheles* behavior. Both models include these parameters as fixed effects: treatment, time since product replacement (and interaction with treatment), week number to account for seasonality within the study period, village (“rural” Nalube compared to “urban” Mapanza), the number of members in the household, and temperature. The hourly occupancy rate was determined by dividing the hourly occupancy rate that was recorded by mosquito collectors by the number of household members. This parameter, alongside hour (to account for hourly host-seeking patterns), and the hourly wind speed were additionally included in hourly models. Random effects were included to account for night-to-night variation (collection date term), cluster-to-cluster variation (cluster term), and within-cluster household-to-household variation (household term nested within cluster). Some parameters were not included in non-*Anopheles* models due to model fits. A series of univariate analyses and two best-fit interaction models (nightly and hourly) were constructed for *Anopheles* behavior, measuring the impacts of measured predictors (such as temperature, relative humidity, moonlight intensity, and household factors) on mosquito activity and SE efficacy. Significant predictors of activity and/or efficacy are presented. Enrollment sizes were determined at the nightly level by simulations based on local historical CDC light trap collection, and an assumption that HLC collections would replicate the CDC-LT collections which were frequently animal-baited, with an assumed 60% reduction in mosquito host-seeking associated with the treatment.

### Ethics statement

This study was approved by the institutional review board at the University of Notre Dame (Protocol #: 18-05-4675) and by the local IRB at Macha Research Trust (IRB #: IRB0007649). All study participant heads of household provided written informed consent for enrollment and product placement. Mosquito collectors and local supervisors provided informed consent to collect mosquitoes and were provided malaria prophylaxis. Study was conducted with approval from the Zambian National Health Research Authority (Ref No. NHRA00001/10/09/2020) and local community leaders. SE products were imported under the Zambia Environmental Management Agency (ZEMA Licence No. LSK/PTS/02412/Z07/2020).

## Results

### Weather conditions

Rain occurred on 12 out of 53 total follow-up collection nights. The occurrence of rain was reported each hour by mosquito collectors in collection forms. Rain was not directly quantified and was often sporadic, with a median of 19.5% of collection hours impacted by rainfall during rainy nights. The mean overnight temperature fell from above 19 degrees in February collections to below 14 degrees in May and June. Relative humidity also dropped in this interval from 95% to 70%.

### Mosquito abundance and identification

A total of 3021 mosquito specimens were collected, of which 1513 (50.1%) were *Anopheles* species. The remaining 1508 (49.9%) were identified as *Culex* species. The most prominent *Anopheles* species as determined by morphology was *An. gambiae s.l.* (n = 1314), with six additional species (total n = 165) each with under 5% total representation and an additional 34 unidentifiable (damaged) specimens (Table 1).

**Table 1:** Raw numbers of mosquitoes captured during follow-up period in all households.

*Anopheles* species	Total number	%
* coustani*	67	4.4
* gambiae s.l.*	1314	86.8
* longipalpis*	43	2.8
* maculipalpis*	1	0.1
* pretoriensis*	6	0.4
* rufipes*	29	1.9
* squamosus*	19	1.3
unknown	34	2.2
(Total)	1513	--
*Culex* sp.		
(Total)	1508	--

### Intervention efficacy

**Fig 3:**
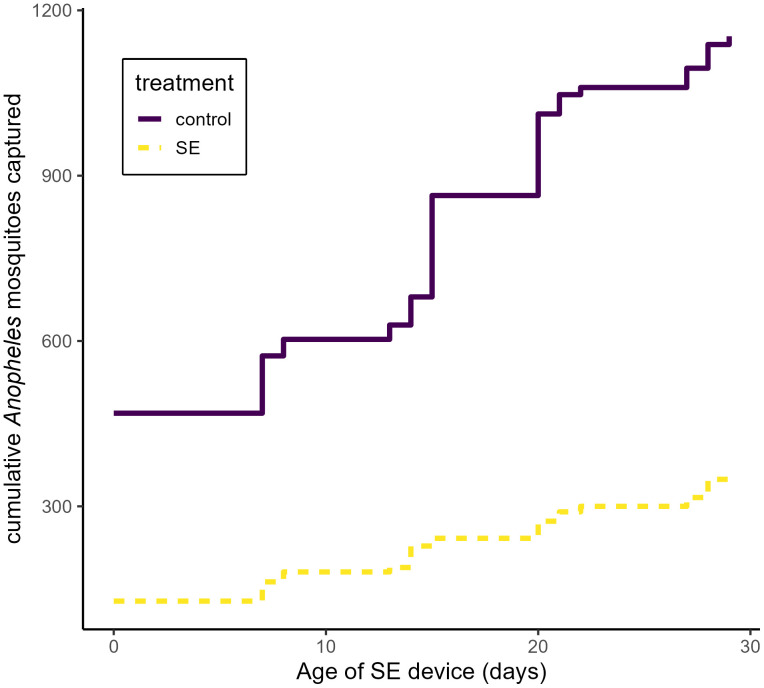
Cumulative number of *Anopheles* mosquitoes captured over the duration of deployment time. Cumulative host-seeking activity is displayed on the y-axis across device ages (x-axis), separated by treatment status. The dotted line indicates kitchens which were provided with the SE device.

**Fig 4:**
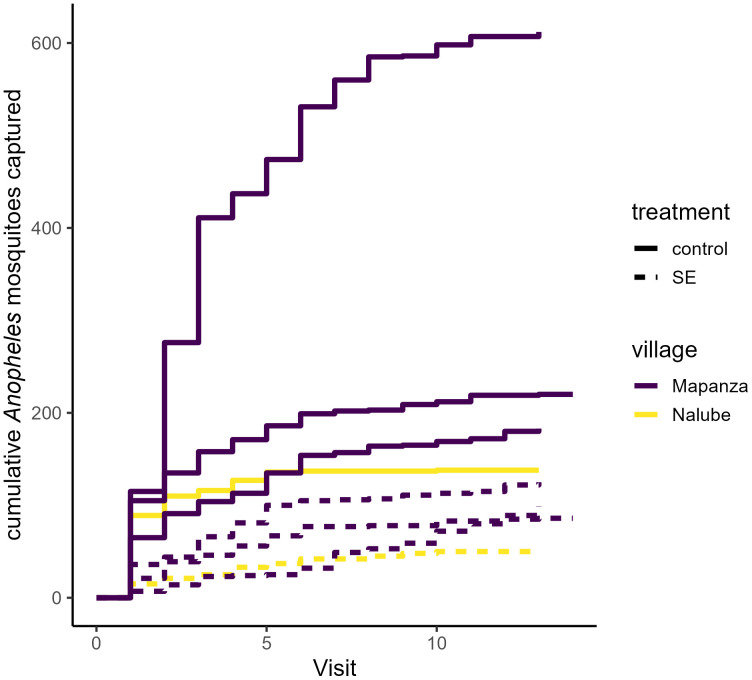
Cumulative number of *Anopheles* mosquitoes by cluster over the study follow-up period. Cumulative host-seeking activity is displayed on the y-axis across weekly visits (x-axis). The line color denotes the collection village (Mapanza and Nalube), with the dashed lines representing clusters utilizing the SE device.

**Fig 5:**
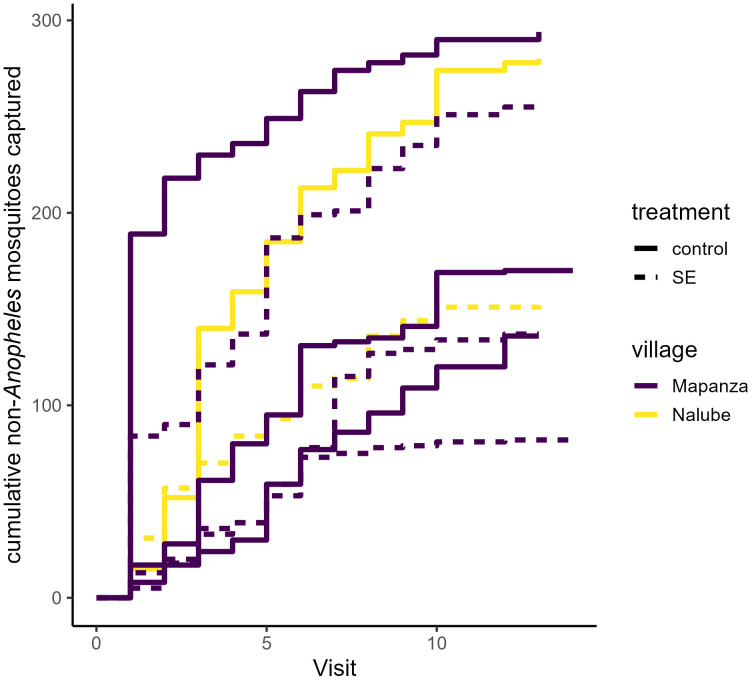
Cumulative number of non-*Anopheles* mosquitoes by cluster over the study follow-up period. Cumulative host-seeking activity is displayed on the y-axis across weekly visits (x-axis). The line color denotes the collection village (Mapanza and Nalube), with the dashed lines representing clusters utilizing the SE device.

A total of 1153 *Anopheles* mosquitoes were captured across all study nights in control kitchens, compared to 360 in kitchens with deployed SE devices (Fig 3). The SE device was associated with significant reduction of measured human landing by *Anopheles* mosquitoes on a nightly (PE (1-RR*100): 69% [[42–84], p < 0.001) and hourly (PE: 74% [51–86], p < 0.001) basis (Table 2). SE efficacy was highest with fresh devices and gradually diminished over their monthlong use (daily RR: 1.03 [1.01–1.05], p < 0.001). Overall *Anopheles* activity was lower in each of the treatment clusters compared to control clusters (Fig 4). A total of 880 *Culex* mosquitoes were captured in control kitchens compared to 628 in treatment kitchens (Fig 5), and their behavior was not significantly impacted by household treatment status throughout the study period (Table 3).

**Table 2:** Negative binomial model coefficients for nightly and hourly models of *Anopheles* behavior during follow-up period.

(A) Nightly model			(B) Hourly model	
	p	FIXED EFFECTS		p
2.44 [1.45–4.10]	**0.001**	(Intercept)	**0.19 [0.11–0.33]**	**< 0.001**
0.31 [0.16–0.58]	**< 0.001**	Treatment (SE)	**0.26 [0.14–0.49]**	**< 0.001**
0.37 [0.30–0.45]	**< 0.001**	Week^#^	**0.36 [0.29–0.43]**	**< 0.001**
NA		Hour^#^	**1.32 [1.21–1.44]**	**< 0.001**
0.31 [0.14–0.69]	**0.004**	Village 2	**0.32 [0.14–0.75]**	**0.009**
0.98 [0.96–1.00]	**0.017**	Device Age	**0.98 [0.96–1.00]**	**0.014**
		**Interactions**		
1.03 [1.01–1.05]	**0.010**	SE: Age	**1.03 [1.01–1.04]**	**< 0.001**
NA		SE: Hour^#^	**0.51 [0.44–0.60]**	**< 0.001**

Fixed effects and interaction term are displayed as the exponentiated coefficient and 95% confidence intervals. Week refers to the epidemiological week of data collection and is included to account for the seasonal decline in abundance during the study. Age refers to the age of the device (in weeks) after first opening (Age 0). Random effects of study night, household, and cluster were included in both models. Degrees of freedom and AIC are provided for each model with the corresponding null model values in parenthesis.

#Variables have been scaled and centered.

**Table 3:** Negative binomial model coefficients for nightly and hourly models of *Culex spp.* behavior during follow-up period.

(A) Nightly model			(B) Hourly model	
	p	FIXED EFFECTS		p
1.65 [1.13–2.41]	**0.010**	(Intercept)	**0.13 [0.09–0.19]**	**< 0.001**
0.76 [0.49–1.19]	0.225	Treatment (SE)	0.77 [0.49–1.19]	0.240
0.36 [0.27–0.47]	**< 0.001**	Visit^#^	**0.35 [0.27–0.46]**	**< 0.001**
NA		Hour^#^	**0.84 [0.77–0.92]**	**< 0.001**

Fixed effects and interaction term are displayed as the exponentiated coefficient and 95% confidence intervals. Random effects are displayed as the exponentiated variance attributed to each parameter. Degrees of freedom and AIC are provided for each model with the corresponding null model values in parenthesis.

#Variables have been scaled and centered.

### Mosquito behavior

**Fig 6:**
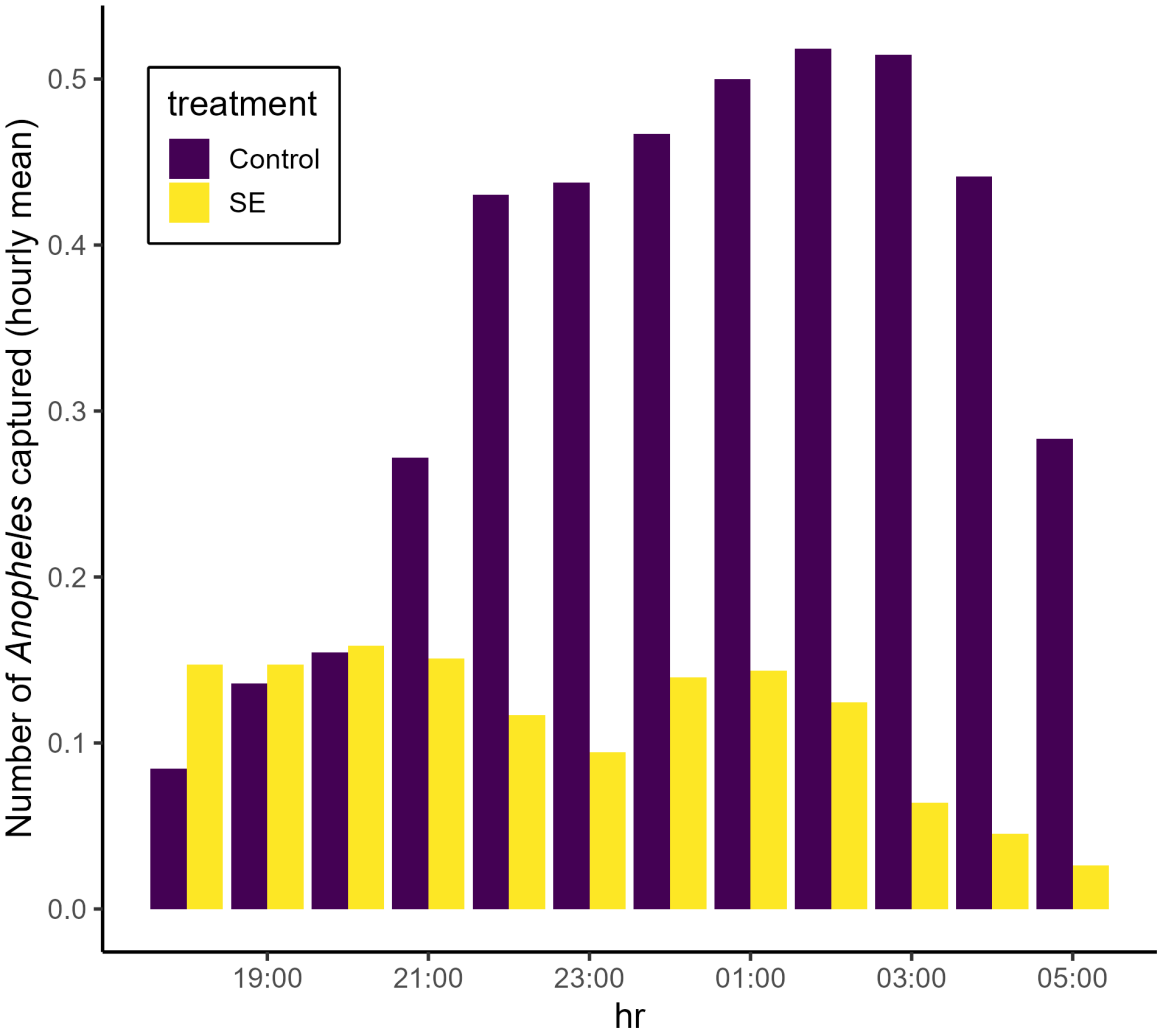
Hourly distribution of *Anopheles* captured per collection hour from 18:00-19:00 to 05:00-06:00 across study follow-up collections, displayed as the hourly mean capture and separated by treatment status.

Mosquito activity declined throughout the study period, with the highest rate of *Anopheles* capture occurring in February (n = 226; 11.9 *Anopheles* per night over 19 collection nights). Fewer mosquitoes were collected in each successive month, with 0.25 *Anopheles* captured per kitchen per night (n = 8 over 32 collection nights) during June, the last month of collection (S1 Table). Models indicate a significant decline in *Anopheles* activity per visit, with additional variation between collection nights modeled as a random effect (Table 2). Collectors in the most active control cluster encountered 1.88 *Anopheles* per kitchen per night, compared to the remaining control clusters which each captured fewer than 0.56 (mean = 0.49) *Anopheles* per kitchen per night (S1 Table). Across all clusters and collection nights, the highest numbers of *Anopheles* were captured between 01:00 and 03:00 (n = 174 per hour). The lowest activity was observed between 18:00 and 19:00 (n = 62) and was generally lowest between the hours of 18:00–22:00 and 04:00–05:00 (Fig 6, S1 Table). *Anopheles* and *Culex* mosquito host-seeking was significantly higher on warmer nights or hours, and significantly lower during hours of higher wind speed (Table 2, Table 3). Host-seeking of *Anopheles* and *Culex* significantly declined throughout the study and there was a significant difference in *Anopheles* activity between villages (Table 2, Table 3).

### Human behavior

**Fig 7:**
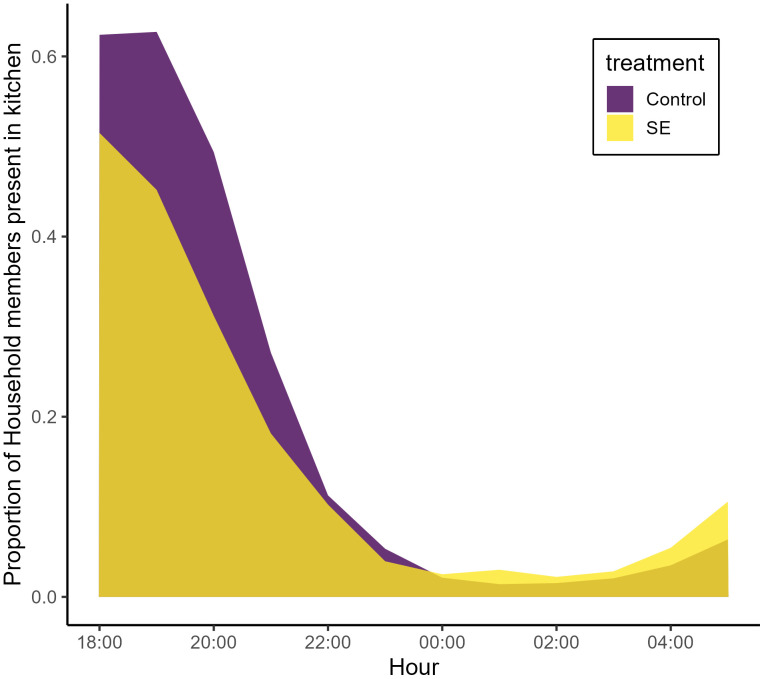
Proportions of households using the kitchen during the indicated hour between 18:00-19:00 and 05:00-06:00, as recorded by mosquito collectors. Treatment status indicated by color.

Hourly survey data of human activity within the kitchens revealed that the average proportion of household members in or near the kitchens was highest in the early evening hours between 18:00 and 21:00. Activity was similar in each hour with medians of 54.3%, 53.3%, and 39.3% of household members in the kitchen space during each of these hours (Fig 7). The number of individuals living in each household was not significantly different between treatment arms (mean: 7.7 (SD: 3.0) individuals per house in the treatment arm, 7.6 (SD:3.0) in the control arm; t(39) = 0.03, p = 0.97), nor was the kitchen size, which was slightly larger in SE structures (mean: 18.4 sq. m (SD = 9.2) compared to 15.8 sq. m (SD = 4.5); t(27) = 1.2, p = 0.26). Over the course of the study, occupancy rates of the kitchen spaces were significantly lower in SE households overall, particularly during the early hours (Fig 6; overall hourly RR: 0.82 [0.79–0.86], p < 0.001). Kitchen occupancy significantly decreased during later hours across treatment arms (p < 0.001). The number of animals residing in the kitchen spaces declined slightly throughout experimental nights (hourly RR: 0.98 [0.98–0.99], p < 0.001), and kitchens enrolled in the treatment study arm reported significantly higher numbers of resident animals (mean number of animals in treatment arm kitchens: 3.5 (SD: 6.4), mean in control arm: 2.0 (SD: 3.2); animal occupancy RR: 1.76 [1.71–1.81], p < 0.001). Presence of fire in the kitchens dropped throughout the night (hourly binomial odds ratio: 0.83 [0.81–0.85], p < 0.001), with no difference in fire usage between treatment arms (p = 0.768).

### Influence of measured factors on mosquito activity and SE efficacy Best-fit models

The full interaction nightly model associated higher nightly *Anopheles* activity with higher temperatures (S2 Table; RR: 2.07 [1.45–2.96], p < 0.001), larger numbers of household residents (RR: 1.36 [1.10–1.67], p = 0.005), and higher proportions of residents in kitchens (RR: 1.20 [1.00–1.44], p = 0.049). More *Anopheles* were active in households with higher localized (5m) NDVI (RR: 1.36 [1.13–1.65], p = 0.002). Activity was lower in SE-protected kitchens during warmer nights (RR: 0.49 [0.38–0.63], p < 0.001) and in more populated (by percentage of household members) kitchens (RR: 0.65 [0.48–0.88], p = 0.005). In the hourly model, higher temperature (RR: 1.80 [1.38–2.36], p < 0.001) and higher values of nearby (5m) vegetation (1.28 [1.04–1.57], p = 0.022) were associated with more *Anopheles* activity overall. Observed activity was lower when a larger proportion of the household members were in the kitchen (RR: 0.76 [0.68–0.84], p < 0.001), during hours with greater moonlight intensity (RR: 0.76 [0.69–0.84], p < 0.001), and when rainfall was observed by collectors (RR: 0.67 [0.47–0.96], p = 0.029). SE efficacy was significantly higher during warmer (RR: 0.54 [0.42–0.68], p < 0.001) and more humid hours (RR: 0.62 [0.49–0.78], p < 0.001). Other relationships between these environmental predictors and mosquito activity/efficacy did not improve the model fit.

### Univariate interactions

Several interactions were not significant predictors of mosquito activity or SE efficacy in the best-fit models but were significant predictors in univariate analysis. Lower mosquito activity was observed on nights with higher humidity (RR: 0.66 [0.49–0.88], p = 0.005) and hours with higher average wind velocity (RR: 0.68 [0.52–0.89], p = 0.005). Higher nightly wind velocity was associated with lower SE efficacy (RR: 1.50 [1.10–2.10], p = 0.008), while hourly efficacy was observed to be higher in hours when rainfall occurred (RR: 0.32 [0.14–0.72], p = 0.018). Higher numbers of household residents overall (RR: 1.80 [1.10–3.00], p = 0.016), kitchen occupants (RR: 1.24 [1.02–1.50], p = 0.03), and the presence of fire (RR: 1.50 [1.10–2.10], p = 0.02) were all associated with lower efficacy. None of these interactions were significant in the best-fit models presented in the previous paragraph.

## Discussion

The results demonstrate that the SE devices were effective in reducing the rate of outdoor *Anopheles* mosquito host-seeking behavior by about 65–70% when deployed in open-walled kitchens of southern Zambia. As the devices aged, their efficacy appeared to wane by approximately 3% per day in this setting, with an estimated efficacy of 20–25% four weeks after opening. These results support previous findings regarding transfluthrin-based spatial emanators, mostly conducted in Tanzania and Southeast Asia [28,33–35,44–46].

The drop-off of efficacy associated with device age has been observed previously, but generally to a lesser degree than observed here [46]. The device age was included with a unit of days after opening to account for minor variation, but was measured generally on weekly intervals (i.e., freshly opened, one week old, etc.). It may be possible to estimate a longevity curve for these devices with more frequent measurements, which could be useful for understanding protection during short (<1–2 weeks) or longer use cases.

Variation in mosquito activity was high between households, with greater random variation attributed to household than to the cluster. The variation was likely due to a variety of local factors, including the location of the household and its surroundings, the attractiveness of the collectors to mosquitoes, the ability of the collectors, and other factors. These factors were assumed to be evenly distributed between the treatment and control study arms. Mosquito behavior varied more between study households than it did between collection nights, and varied slightly by hour, according to the mixed effect models. The variance associated with household ID was roughly half the magnitude of the estimated coefficient for the treatment effect in the final models. Transfluthrin volatility increases with ambient temperatures, and the manufacturer recommended temperatures above ~24 degrees C for the tested product. Temperatures were cooler than this recommendation throughout the study period – especially later – with the SE estimated to have greater efficacy during the comparatively warmer periods of the study.

Mosquito activity was highest during the earliest mosquito collections and declined throughout the follow-up period. The initial mosquito collections occurred near peak transmission conditions in the area, with the traditional season generally beginning with the early rains in November or December. Rain over long periods of collection nights was rare; only about one in five nights experienced rainfall, usually for a few hours per night. The nightly temperature remained consistent for the first three collection months but dropped quickly near the end of April through the end of the collection period. The nightly behavioral trend is driven by morphologically identified *An. gambiae* s.l. mosquitoes which represent nearly all captured *Anopheles* mosquitoes and are likely *An. arabiensis* based on historical collection data [12,20,21,47]. The other traditionally dominant species in the area, *An. squamosus*, was considerably less prevalent in collections than expected based on previous mosquito surveys.

Overall, mosquitoes were more frequently captured in households with more people but not more animals, evidence that the captured mosquitoes in this study were anthropophilic or otherwise attracted to stronger host-seeking cues (i.e., odors) of larger households, although this study did not assess host preference or account for the presence of nearby animals outside the kitchens. Mosquito activity appeared to peak during later hours, particularly in control kitchens. The efficacy of the SE is high during these hours, but there is an apparent lack of efficacy in the first three hours of the night. It is possible that this effect is due to some unmeasured aspect of the study that was unbalanced between arms, but another possibility is that the odors associated with cooking and smoke (and the increased number of nearby humans during these hours) may mask or overcome the chemical repellency of transfluthrin. Limited evidence suggests that smoke inhibits mosquito activity, but it’s impact on transfluthrin efficacy is unknown [48,49]. Control kitchens were occupied at higher rates during these hours, further convoluting the difference in host-seeking rates since they may have diverted bites from mosquito collectors. This may reflect conscious or unconscious decisions by household members to avoid the SE devices. The control kitchens did contain placebo devices, and no adverse effects were reported by study participants. The hourly difference in efficacy may also be associated in some way with this difference in human activity (i.e., more household members leading to fewer mosquitoes captured by collectors in control kitchens). The hourly dynamic was not observed in two field studies of the SE device when deployed in sleeping structures in Sumatra, Indonesia, and it has direct implications on this use-case of the SE given the overlap of human and mosquito behavior in the kitchen during these hours [39,40]. The overlap of human behavior and mosquito activity is an important element in evaluating interventions, and further work could elucidate the dynamics observed here to determine which factors are driving lack of efficacy and potential transmission in this early evening period. Human behavioral data was secondary analysis for this study, but a properly powered field trial could better determine the overlap of human occupancy and mosquito behavior, and how interventions such as spatial emanators influence this dynamic.

The overall nightly and hourly SE efficacy observed in this study has been observed previously in studies of the SE device. Studies of other devices containing transfluthrin support this evidence and have shown that transfluthrin is effective towards a variety of *Anopheles* species, including those with insecticide resistance [28,32–37,44,46]. A semi-field study conducted in Macha two years prior to this field study showed the devices were effective towards a lab strain of *An. gambiae* s.s. [38]. That study contained additional endpoints which indicated that the devices had a small impact on mosquito survivability. A field study and separate field based rotational study of the SE in Sumatra yielded similar estimates of efficacy in a warmer and wetter transmission environment (Manuscripts under review, Malaria Journal). The evidence is mixed but has generally supported a slow decline in efficacy over time in all conditions. Further work could delve into the contribution of environmental conditions to efficacy and optimal deployment conditions.

Future studies of the SE device can continue to confirm the efficacy of the device across transmission environments that contain a variety of *Anopheles* species. Measuring the volatizing transfluthrin active ingredient would aid in quantifying the longevity of the devices, and additional time points between the weekly intervals would also be informative. As evidence of overall field efficacy mounts, it may be beneficial to conduct field studies which can quantify entomological impacts beyond those which are currently considered. This includes diversion or community effects via mortality or altered fecundity, effects on mosquito behavioral patterns, the size of the area of effect, and direct comparison of human behaviors, or potential other factors which may impact efficacy in the early evening hours (ex. cooking). Measuring epidemiological outcomes such as parasite burden or disease incidence rates requires large scale studies – especially in near-elimination settings considering the small number of cases observed, but such studies provide quality evidence of the potential of these devices as disease intervention tools [50].

## Supporting information

S1 Table Raw numbers of *Anopheles (*left two columns) and non-*Anopheles* (right two columns) mosquitoes captured during the study follow-up period. Both groups are separated into columns of SE and control mosquitoes. Data is separated by cluster, month of capture, and hour. For monthly numbers, the number of collection nights per treatment arm is indicated in parenthesis.
(DOCX)

S2 Table Exponentiated full interaction model coefficients for nightly and hourly models of *Anopheles* mosquito behavior during follow-up period in study kitchens. Fixed effects and interaction term are displayed as the exponentiated coefficient and 95% confidence intervals. Random effects are displayed as the exponentiated variance attributed to each parameter. Degrees of freedom and AIC are provided for each model with the corresponding null model values in parenthesis. ^#^ Variables have been scaled and centered.
(DOCX)

S1 Data Study Data. (ZIP)
