# Supplementary material for: Efficacy of a volatile pyrethroid spatial emanator (SE) in reducing Anopheles host-seeking in outdoor kitchens in Southern Zambia
Source: PLoS One. 2025 Nov 6;20(11):e0335514. doi: 10.1371/journal.pone.0335514 (PMC12591450; doi:10.1371/journal.pone.0335514)
Supplement: S1 Table — Both groups are separated into columns of SE and control mosquitoes. Data is separated by cluster, month of capture, and hour. For monthly numbers, the number of collection nights per treatment arm is indicated in parenthesis. (DOCX) [file pone.0335514.s001.docx]

Table S1

|  | *Anopheles* sp. | | Non-*Anopheles* sp. | |
| --- | --- | --- | --- | --- |
| Cluster | **VPSR** | **Control** | **VPSR** | **Control** |
| 1 | 50 | 138 | 152 | 280 |
| 2 | 99 | 612 | 137 | 294 |
| 3 | 125 | 183 | 257 | 136 |
| 4 | 86 | 220 | 82 | 170 |
| Total (Cl. mean) | **360 (90)** | **1153 (288)** | **628 (157)** | **880 (220)** |
| Month | **VPSR** | **Control** | **VPSR** | **Control** |
| Feb (30) | 22 | 204 | 36 | 204 |
| Mar (75) | 173 | 681 | 277 | 314 |
| Apr (75) | 88 | 204 | 251 | 232 |
| May (90) | 73 | 60 | 62 | 130 |
| Jun (15) | 4 | 4 | 2 | 0 |
| Hour | **VPSR** | **Control** | **VPSR** | **Control** |
| 18 | 39 | 23 | 46 | 64 |
| 19 | 39 | 37 | 76 | 61 |
| 20 | 42 | 42 | 45 | 74 |
| 21 | 40 | 74 | 48 | 74 |
| 22 | 31 | 117 | 57 | 74 |
| 23 | 25 | 119 | 73 | 103 |
| 00 | 37 | 127 | 80 | 78 |
| 01 | 38 | 136 | 62 | 87 |
| 02 | 33 | 141 | 56 | 82 |
| 03 | 17 | 140 | 36 | 93 |
| 04 | 12 | 120 | 29 | 60 |
| 05 | 7 | 77 | 20 | 30 |
