# Supplementary material for: Efficacy of a volatile pyrethroid spatial emanator (SE) in reducing Anopheles host-seeking in outdoor kitchens in Southern Zambia
Source: PLoS One. 2025 Nov 6;20(11):e0335514. doi: 10.1371/journal.pone.0335514 (PMC12591450; doi:10.1371/journal.pone.0335514)
Supplement: S2 Table — Fixed effects and interaction term are displayed as the exponentiated coefficient and 95% confidence intervals. Random effects are displayed as the exponentiated variance attributed to each parameter. Degrees of freedom and AIC are provided for each model with the corresponding null model values in parenthesis. # Variables have been scaled and centered. (DOCX) [file pone.0335514.s002.docx]

Table S2

| (A) Nightly model | | Modeled endpoint | (B) Hourly model | |
| --- | --- | --- | --- | --- |
| Negative binomial | | Model family | Negative binomial | |
|  | p | **FIXED EFFECTS** |  | p |
| 1.56 [0.98 – 2.46] | 0.058 | (Intercept) | **0.15 [0.08 – 0.27]** | **< 0.001** |
| 0.55 [0.31 – 0.99] | **0.049** | Treatment (VPSR) | **0.44 [0.22 – 0.88]** | **0.020** |
| 0.38 [0.26 – 0.56] | **< 0.001** | Visit^#^ | **0.42 [0.31 – 0.57]** | **< 0.001** |
| NA |  | Hour^#^ | **1.15 [1.00 – 1.32]** | **0.047** |
| 0.32 [0.15 – 0.69] | **0.004** | Village 2 | **0.35 [0.14 – 0.87]** | **0.023** |
| ----- |  | Device Age | **0.98 [0.96 – 0.99]** | **0.026** |
| 2.07 [1.45 – 2.96] | **< 0.001** | Temperature | **1.80 [1.38 – 2.36]** | **< 0.001** |
| 0.69 [0.52 – 0.92] | **0.010** | Relative humidity | **1.23 [0.95 – 1.58]** | **0.110** |
| NA |  | Rainfall | **0.67 [0.47 – 0.96]** | **0.028** |
| ----- |  | Moonlight intensity | **0.76 [0.69 – 0.84]** | **< 0.001** |
| 1.36 [1.10 – 1.67] | **0.005** | HH members (#) | ----- |  |
| 1.20 [1.00 – 1.44] | **0.049** | Proportion HH outside | **0.76 [0.68 – 0.83]** | **< 0.001** |
| 1.36 [1.13 – 1.65] | **0.002** | May NDVI (5m) | **1.39 [1.15 – 1.68]** | **0.001** |
|  |  |  |  |  |
|  |  | **Interactions** |  |  |
| ----- |  | VPSR : Age | **1.02 [1.00 – 1.03]** | **0.042** |
| NA |  | VPSR : Hour^#^ | **0.52 [0.42 – 0.65]** | **< 0.001** |
| 0.49 [0.38 – 0.63] |  | VPSR : Temp^#^ | **0.54 [0.43 – 0.68]** | **< 0.001** |
| ----- |  | VPSR : RH^#^ | **0.62 [0.49 – 0.78]** | **< 0.001** |
| 0.65 [0.48 – 0.88] |  | VPSR : Prop. HH outside | ----- |  |
|  |  |  |  |  |
|  |  | **RANDOM EFFECTS** |  |  |
| 1.17 |  | Collection Date (Int.) | **1.32** |  |
| 1.10 |  | Cluster (Int.) | **1.14** |  |
| 1.24 |  | Cluster/Household (Int.) | **1.49** |  |
|  |  |  |  |  |
| 522 (536) |  | Degrees freedom | **6424 (6443)** |  |
| 1744 (2090) |  | AIC | **5496 (6946)** |  |
